# Mechanical oscillations enhance gene delivery into suspended cells

**DOI:** 10.1038/srep22824

**Published:** 2016-03-09

**Authors:** Z. L. Zhou, X. X. Sun, J. Ma, C. H. Man, A. S. T. Wong, A. Y. Leung, A. H. W. Ngan

**Affiliations:** 1 Department of Mechanical Engineering, The University of Hong Kong, Pokfulam Road, Hong Kong, P.R. China; 2 Department of Medicine, The University of Hong Kong, Pokfulam Road, Hong Kong, P.R. China; 3 School of Biological Sciences, The University of Hong Kong, Pokfulam Road, Hong Kong, P.R. China

## Abstract

Suspended cells are difficult to be transfected by common biochemical methods which require cell attachment to a substrate. Mechanical oscillations of suspended cells at certain frequencies are found to result in significant increase in membrane permeability and potency for delivery of nano-particles and genetic materials into the cells. Nanomaterials including siRNAs are found to penetrate into suspended cells after subjecting to short-time mechanical oscillations, which would otherwise not affect the viability of the cells. Theoretical analysis indicates significant deformation of the actin-filament network in the cytoskeleton cortex during mechanical oscillations at the experimental frequency, which is likely to rupture the soft phospholipid bilayer leading to increased membrane permeability. The results here indicate a new method for enhancing cell transfection.

Although a number of biochemical transfection methods, such as cationic liposomes^1^, cationic polymer^2^, calcium phosphate^3^ and virus based methods^4^, have been developed for drug or gene delivery, such methods are only effective for adherent cells since the substrate provides a stable environment to increase the chance of contact between the cells and reagents. When these methods are applied to non-adherent cells suspended in culture medium, they may not work, or the transfection efficiency is very low^5^,^6^,^7^. On the other hand, physical methods to mediate drug delivery have also been attempted. Transfection by microinjection^8^ and AFM manipulation^9^ have been reported during the past few decades, but these are essentially single-cell manipulation methods which are unsuitable for transfecting cells in batches. Pressure elevation or suction was found to mediate the insertion of naked DNA and oligonucleotide into neointimal, medial and adventitial cells of rabbit carotid arteries^10^, rat and human cardiovascular tissues^11^, and naked plasmid DNA and siRNAs into mice tissue cells 12, but like biochemical means, such methods are not effective for suspended cell types for which pressure changes are not easy to be induced. Electroporation has also been used to deliver genetic materials into certain cell types which are hard to be transfected otherwise^6^,^13^,^14^, but the method normally jeopardizes viability and possibly results in ion imbalance that may lead to improper cell functions^15^.

Therefore, effective and convenient methods for transfecting non-adherent or trypsinized adherent cells in batch quantities are yet to be developed. In this study, we show that mechanical vibrations for short durations at suitable frequencies can lead to membrane disturbances, so that nanoparticles and oligonucleotides can be easily delivered into the cell, while the cell viability is not affected by the vibrations themselves. Specifically, flow cytometry and Western Blot analyses were used to show that siRNAs can be efficiently transfected this way into K562 leukemia cells and become functional in gene inhibition. The mechanism of membrane disturbances by mechanical vibrations is also investigated by finite-element simulation of the cytoskeleton cortex. This study shows that mechanical oscillations can facilitate *in vitro* cell transfection, with the immense prospect of being applicable to a wide range of cell types in batch quantities.

## Results

### Determination of mechanical oscillation condition for nanoparticle transfection

**Figure 1:**
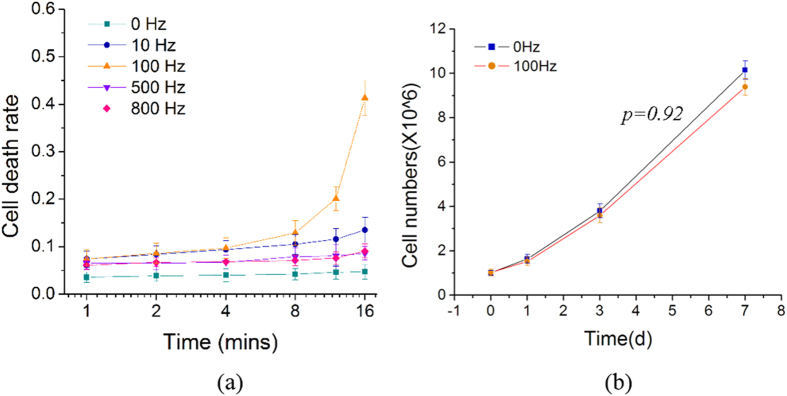
(**a**) Death rate of K562 cells exposed to mechanical oscillations at different frequencies (0, 10, 100, 500 and 800 Hz) and 10 volt amplitude for 0, 1, 2, 4, 8, 12, 16 mins; (**b**) Death rate of K562 cells exposed to oscillations at 100 Hz and 10 volt amplitude for 4 mins following by culture for 1, 3 and 7 days, measured using hemocytometry.

K562 myelogenous leukemia cells in their culture medium were placed inside EP tubes fixed onto the motion bar of a sine-wave generator (see Figure S1 in Supplementary Material). Figure 1(a) shows the death rate of these cells after exposure to mechanical oscillations at different frequencies (0, 10, 100, 500 and 800 Hz) at 10 volt input amplitude for the generator, for different durations (1, 2, 4, 8, 12 and 16 mins). The death rates of K562 cells, measured by hemocytometry based on trypan blue staining, show a gradual increase at increasing oscillation duration for all four frequencies tested (10, 100, 500 and 800 Hz). The death rate of K562 cells oscillated at 100 Hz increases much more significantly for durations longer than ~10 mins, compared to other groups. Two tailed chi-square tests (two populations) showed that the death rates of the K562 cells subjected to oscillations at 100 Hz are significantly different (*p* < 0.01) from the control group, namely, the group without oscillations (0 Hz), for all durations tested, and also different (*p* < 0.05) from the other three groups vibrated at 10 Hz, 500 Hz and 800 Hz, for durations longer than 8 mins. The death rate of the control group but nevertheless still fixed to the sine-wave generator in the same way as other groups, is not higher than 4.8%. The results show that cells subjected to prolonged (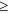 10 mins) oscillations at 100 Hz can receive more damage as has been reported before^16^, but this is not the case for short durations of oscillations. In order to test the invisible damage possibly induced by the mechanical oscillations, the viability of K562 cells, which were exposed to oscillations at 100 Hz and 10 volt amplitude for 4 mins, and later cultured for 1, 3 and 7 days, was measured as shown in Fig. 1(b). At 100 Hz for 4 mins, Fig. 1(a) shows that the death rate is only slightly higher than that at other frequencies for the same duration, and Fig. 1(b) indicates that very similar amounts of healthy cells could be cultured after 1, 3 and 7 days, between the group receiving no oscillation, and that oscillated at 100 Hz for 4 mins. Although the viability/number of cultured cells after exposure to 100 Hz oscillations was slightly lower than the control group, the difference is insignificant according to two tailed chi-square tests (two populations). The results therefore show that the present oscillations for a few minutes do not affect the viability of K562 cells.

**Figure 2:**
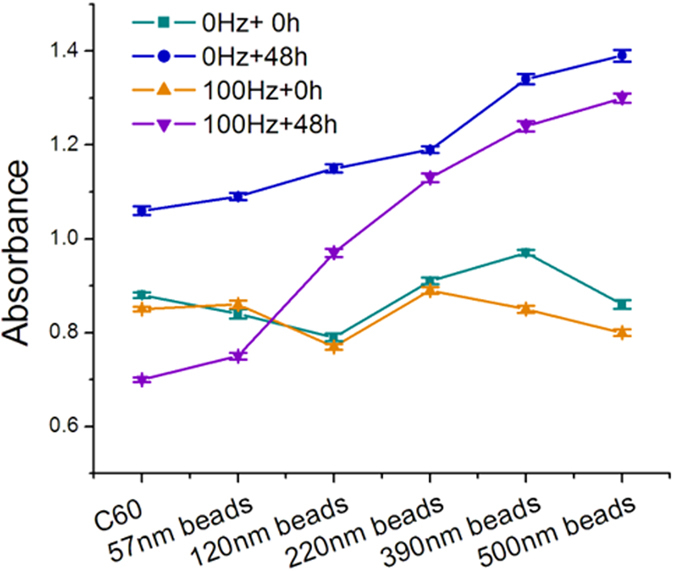
MTT measurements of K562 cells oscillated with nano-particles of different sizes, showing smaller particles producing higher cytotoxicity after oscillations at 100 Hz and 10 volt amplitude for 4 mins.

In Fig. 2, the viability of K562 cells, which were mixed with different nano- and micro-particles (C60 molecules, and polystyrene beads of diameter 57 nm, 120 nm, 220 nm, 390 nm and 500 nm), then oscillated at 100 Hz for 4 mins followed by culturing for 48 hours, was measured by the MTT assay. The results show that after incubation for 48 hours with or without the vibration treatment, the cell viability decreases gradually from those mixed with larger particles to smaller ones. It can be seen that after 48 hours of culture, cells which were mixed with C60 particles or 57 nm diameter polystyrene beads and vibrated for 4 mins, exhibit lower viability/absorbance than the cells mixed with the same types of particles but without receiving the vibration treatment, and also even lower viability than the cells mixed with the same particles and vibrated in the same way, but without the post-vibration culture. This indicates that C60 and the 57 nm diameter polystyrene beads inhibit cell proliferation after the cells were subjected to the mechanical oscillations. The results here suggest that, while these two types of nano-particles can penetrate through the K562 cell membrane to produce cytotoxicity and interfere cellular activity and viability^17^, their ability to do so is significantly enhanced by the vibration treatment. The morphologies of K562 cells mixed with different particles as observed under the Scanning Electron Microscope after dehydration treatment are shown in Figure S2 in the Supplementary Material.

### Implementation and Evaluation of siRNAs Transfection

**Figure 3:**
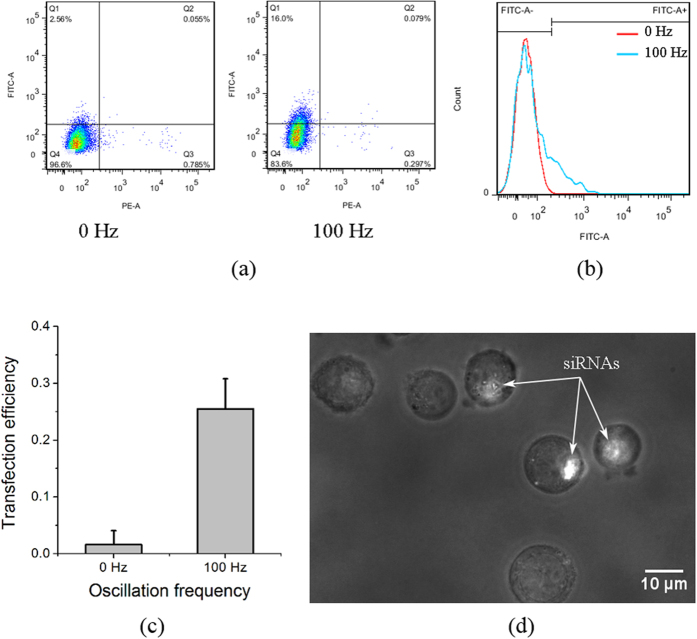
Flow-cytometry results of actin-siRNAs transfection, showing that fluorescence labelled siRNAs penetrated into the cells after mechanical oscillations. (**a**) Flow cytometry graph of FITC-A vs PE-A, indicating the actin-siRNAs transfection efficiency and death rate of K562 cells with and without subjecting to mechanical oscillations; (**b**) Flow cytometry graph of the siRNAs transfection efficiency of K562 cells with and without subjecting to mechanical oscillations; (**c**) Histogram of actin-siRNAs transfection efficiency of K562 cells with and without subjecting to mechanical oscillations; the error bars indicate standard deviations of three measurements; (**d**) Optical image showing FITC labelled actin-siRNAs transfected in the K562 cells.

To study how the transfection of K562 cells by siRNAs can be affected by mechanical oscillations, cells mixed with the actin-targeted FITC labelled siRNAs were exposed to mechanical oscillations at 100 Hz and 10 volt amplitude for 4 mins. Figure 3(a) shows the flow cytometry intersection graph (FITC vs PE), indicating the transfection efficiency (FITC) and death rate (PE) of the K562 cells with or without receiving the oscillations. By comparing the PE data between the 0 Hz and the 100 Hz groups, it can be seen that the death rate of the main population of K562 cells is not affected by the 4-minute oscillations at 100 Hz and 10 volt amplitude, after 24-hour cell culture and recovering. However, the FITC data in Fig. 3(a,b) show that FITC labelled siRNAs were transfected into the K562 cells after the mechanical oscillations. Figure 3(c) shows that the transfection efficiency is up to 30%. It is expected that the transfection efficiency can be varied by the changing the oscillations especially their duration, but the death rate may also change as well. Figure 3(d) shows an optical image captured in a dim condition with the purpose to highlight the fluorescence of the FITC labelled siRNAs. It can be clearly seen that siRNAs were successfully transferred into the cells after the mechanical oscillations.

**Figure 4:**
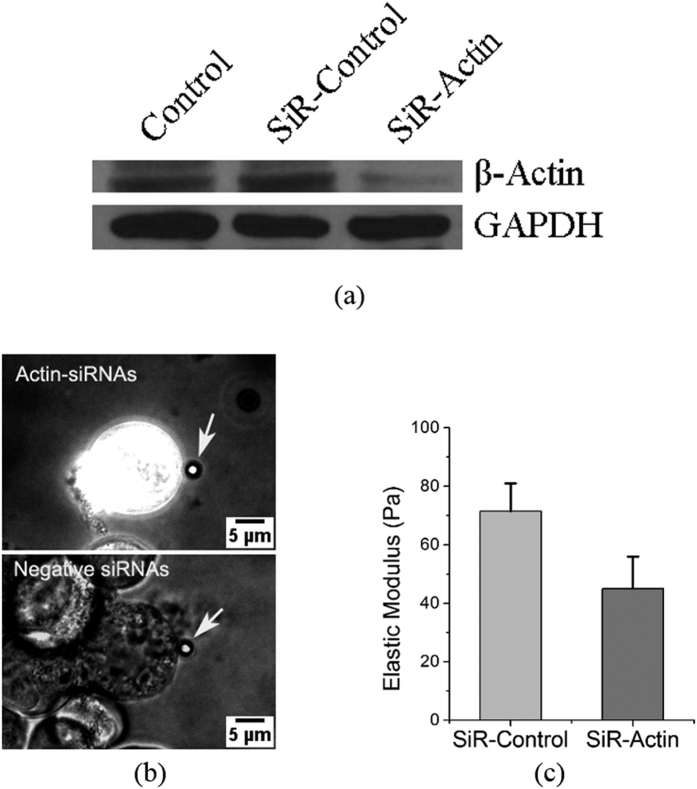
(**a**) Western Blot results showing designed SiR-actin inhibits the β-actin protein expression significantly. Control: K562 cells without exposing to mechanical oscillations; SiR-control: K562 cells mixed with the negative siRNAs sequence and exposed to mechanical oscillations; SiR-Actin: K562 cells mixed with the actin-siRNAs sequence and exposed to mechanical oscillations; (**b**) Optical images of indentation by optical tweezers manipulation, captured at 488 nm excitation wavelength in a dim condition. The white arrows indicate the 2.5 μm polystyrene indenter beads trapped by laser; (**c**) Histogram showing the measured elastic moduli of K562 cells transfected with negative control of siRNAs and FITC labeled actin siRNAs. The error bars indicate standard deviations of 7 measurements.

Western Blot analysis was used to examine the working effect of the actin siRNAs after transfection into K562 cells under the assistance of mechanical oscillations. In Fig. 4(a), the primary antibody of the housekeeping gene GAPDH is used as the inner control. The similar amounts of GAPDH protein in the three groups indicate that the total amount of protein of the three loading samples was well quantified by the DC protein assay kit, implying that it is valid for the comparison of the targeted/tested groups. The protein expression in the actin siRNAs group (SiR-actin) is significantly lower than the control (K562 cells without exposed to mechanical oscillations) and the SiR-control (K562 cells mixed with the negative siRNAs sequence and exposed to the same mechanical oscillations), indicating that the actin siRNAs exhibit an obvious inhibition effect on the actin filament expression while the control and the SiR-control do not show such an inhibition effect. The fact that siRNA transfection inhibits actin filament expression was also confirmed by elastic-modulus measurement using pico-indentation by optical tweezers. Figure 4(b,c) show that the elastic moduli of K562 cells transfected with the negative siRNAs control was measured to be 71.5 Pa, which is similar to the modulus of 74.6 Pa of normal K562 cells without any transfection^18^, but is significantly higher than the measured value of 45.0 Pa of K562 cells transfected with actin siRNAs, indicating that the K562 cells transfected with actin siRNAs are obviously softer than those transfected with the negative siRNAs. Two-tailed Chi-square tests (two populations) showed that the elastic moduli of the two groups transfected with actin siRNAs and the negative control of siRNAs are significantly different (*p* < 0.01).

**Figure 5:**
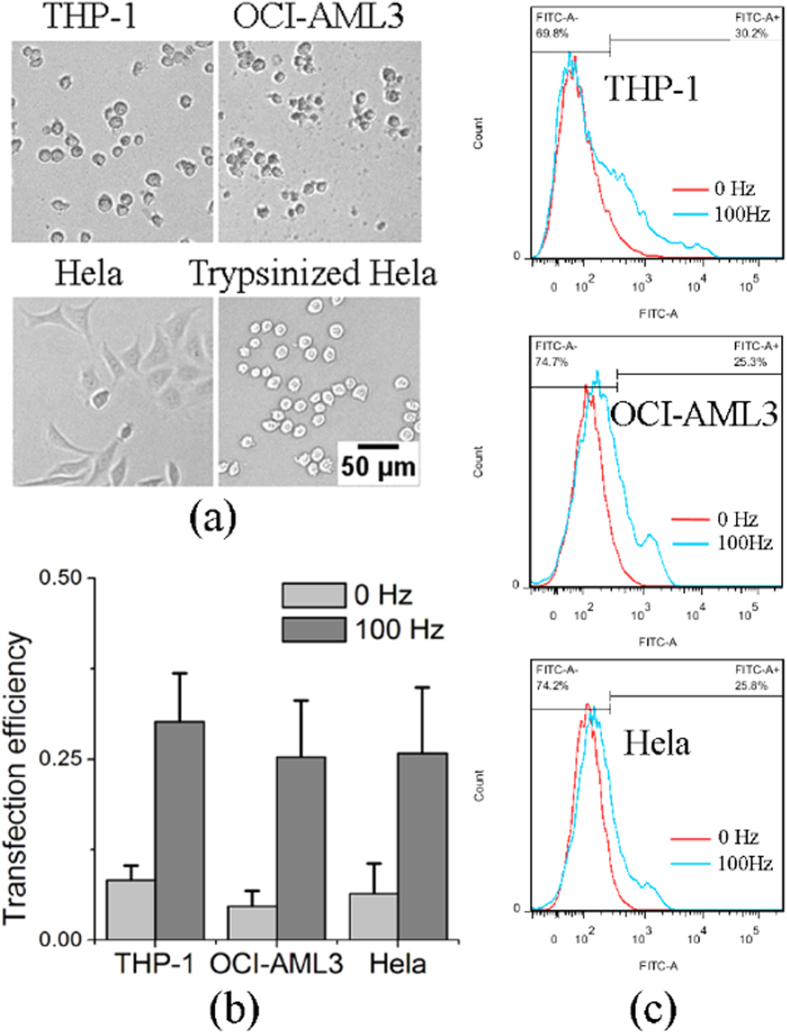
Transfection efficiency of leukemia cell lines THP-1, OCI-AML3 and trypsinized adherent cell line Hela measured by flow cytometey. (**a**) Optical images of the three non-adherent and adherent cell types. Scale bars indicate 50 μm for all images. (**b**) Histogram of actin-siRNAs transfection efficiency of the three cell types with and without subjecting to mechanical oscillations; the error bars indicate standard deviations of three measurements. (**c**) Flow cytometry graph of the siRNAs transfection efficiency of the three cell types with and without subjecting to mechanical oscillations.

In addition to K562 cells, non-adherent leukemia cell lines THP-1 and OCI-AML3, and adherent cell line Hela, were also studied. Figure 5 shows that the transfection efficiency of these cell types with FITC labelled siRNAs as by flow cytometry is increased by mechanical oscillations. Hela cells are adherent cells but they were detached by trypsin into spherical, non-adherent cells before cell transfection, as shown in Fig. 5(a). The results in Fig. 5(b,c) show that mechanical oscillations can enhance the siRNAs efficiency of the different cell types including adherent cell types.

### Mechanism of nanoparticle delivery into cells in modeling

**Figure 6:**
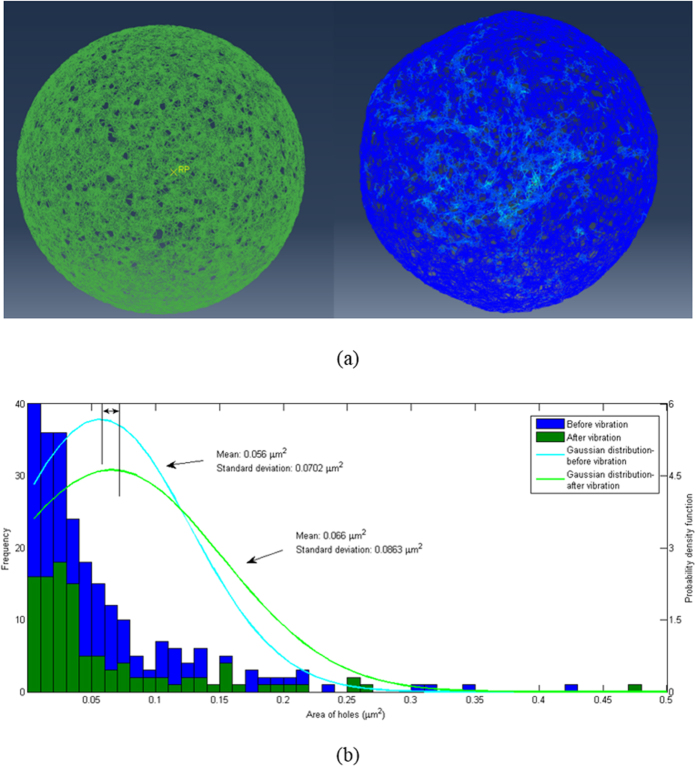
Finite-element simulation of the cytoskeleton cortex. (**a**) Typical simulated configurations of the cortex structure before (left) and after (right) 5 cycles of vibration. An attached movie shows the simulated oscillations. (**b**) Frequency plots of the pore areas on the cortex layer, and fitted Gaussian distributions, before and after 5 cycles of vibration.

The cell membrane is composed of a phospholipid bilayer supported by the cytoskeleton cortex, and so more insights need to be gained as to how exactly mechanical vibrations can disrupt the membrane. The leukemia cells studied in this work were of a spherical shape, and unlike adherent cells the shape of which is determined principally by the focal adhesions with the extracellular matrix, the spherical shape of suspended cells is evidently supported by the cortex layer of the cytoskeleton underneath the plasma cellular membrane. The phospholipid bilayer of the cell membrane is known to be soft with bending stiffness of 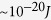
19 which contributes little to the bending stiffness of the membrane of K562 cells of 
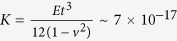
 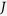, where t ~0.2 *μm* is the thickness of the cell membrane, 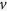 is Poisson ratio (~1/3) and *E* = 74.62 Pa is the elastic modulus measured by optical tweezers indentation experiments^18^. Since the phospholipid bilayer contributes little to the rigidity of the membrane, if oscillatory motions of the cell are to result in enhancement of drug insertion, the more rigid cytoskeleton cortex will have to be mechanically deformed, which may then rupture the attaching plasma membrane to allow the penetration of the nano-materials. To understand the vibration-induced penetration of nano-materials, the key is therefore an understanding on how the cytoskeleton cortex can be deformed by mechanical vibrations. To achieve this goal, a finite-element model for the cytoskeleton cortex was constructed (see Supplementary Material for details). The cortex layer of the cytoskeleton is modeled as a shell of strut-element network of thickness 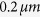
20 on the surface of a spherical cell of radius 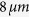
21. 11600 strut elements which represent the actin filaments are present in the cortex layer, and their average length is 0.3 μm. To simulate the mechanical vibrations of the cell, a nodal point on the outer surface of the cortex layer was given an oscillatory motion of amplitude 0.4 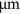 at 100 Hz frequency along a tangential direction. Inertia effects of the intracellular components and filament properties were chosen according to realistic conditions, as described in the Supplementary Material. Figure 6(a) compares the typical configurations of the cortex before and after 5 cycles of simulated vibration. Figure 6(b) illustrates the distributions of the pore areas on the cortex layer, the quantification of which is explained in the Supplementary Material. It can be seen that the oscillations cause the mean pore area to enlarge from the initial value of 0.056 *μm*^2^ to 0.066 *μm*^2^, and the standard deviation also increases from initially 0.0702 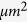 to 0.0863 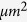. The simulations here show that during forced oscillation at 100 Hz, the cortex deforms significantly, and the pores on it become significantly larger due to the stretching of the actin network around them.

## Discussion

The present results indicate clearly that mechanical oscillations can enhance the delivery of nanomaterials into the cell. The mechanism is likely that the vibrations disturb the cell membrane, thereby increasing the permeability of the membrane to enhance transfection. One question that needs to address is: how does the forced opening of the pores in the cytoskeleton cortex affect the phospholipid bilayer on top, which has to be ruptured in order to allow insertion of nanomaterials. As mentioned above, the lipid bilayer is 3 orders of magnitude less rigid than the cortex. Fischer^22^ studied the slipping of lipids within a lipid layer due to thermally induced fluctuations, by comparing the relaxation time of lateral pressure difference due to inter-lipid friction, with the experimentally observed decay time of membrane shape changes. According to his results, the estimated relaxation time for lateral pressure gradients due to inter-lipid friction is 32 *ns*, 34 *ns* and 35 *ns* for the three lowest modes respectively, which are 9 orders of magnitude lower than the observed decay time of 2.6 s, 0.7 s, and 0.4 s respectively for membrane shape changes^22^. This indicates that the lipids have enough time to slip during the fluctuations. However, when the connection of lipid layers with the cortex is taken into consideration, the coefficient of viscous friction will be increased tremendously. It has been reported that the 2-D viscosity for cytoskeleton attached membrane is about 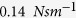 23, which is 8 orders of magnitude higher than the value for isolated lipid layers used in Fischer’s work. Multiplying the ratio of viscosities for attached and isolated lipid layer to Fischer’s estimated relaxation time, the relaxation time will increase to 3.2 s, 3.4 s, and 3.5 s for the three lowest modes due to the increased friction from the attachment. Now the relaxation time is comparable to, and even longer than, the decay time for shape changes, indicating that during membrane shape changes the lipids can hardly slide against the cortex. All these amount to say that during vibrations, the lipid bilayer should be well attached to the cortex, although it is too soft to affect the mechanical deformation of the cortex, which is governed solely by the elasticity of the actin-filament network and the inertia effects of the intracellular components. Since the lipid bilayer is very soft, the deformation of the cortex layer during vibrations should simply rupture it locally, thus allowing enhanced insertion of nano-materials into the cell. The mean simulated pore sizes in the cortex of 0.056 *μm*^2^ initially and 0.066 *μm*^2^ during vibrations (Fig. 6(b)) correspond to linear sizes of 236 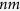 and 257 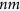 respectively, which are within the size range of the nano-particles studied experimentally in this work. The results in Fig. 5 show that the original suspended Hela cells turn into a spherical shape after detachment by trypsin from the substrate, and the spherical shape suggests that the organization of the cortical cytoskeleton becomes similar to the suspended leukemia cells. Thus, the proposed mechanism of transfection enhancement by mechanical oscillations should also be applicable to the trypsinized Hela cells.

As mentioned previously, physical methods for mediating the delivery of exogenous materials into cells and tissues tend to be simpler and more efficient in implementation, and can even produce more direct transfection outcomes^24^, than biochemical methods which normally need the assistance of certain cellular and subcellular activities such as endocytosis^25^, electrostatic interactions and fusion with the plasma membrane via the lipid moieties of the liposome^26^, as well as the process of host cells recognized and transfected by virus vectors in virus-mediated transfection^27^. Biochemical methods usually require more time for the transfection to succeed since the exogenous material delivery process requires the above-mentioned cellular and subcellular activities to take place. In addition, stricter transfection factors than physical methods, such as the nucleic acid/chemical ratio, solution pH and cell membrane conditions, are required for biochemical methods^7^. Using mechanical vibrations to enhance drug/gene delivery into mammal cells has the merit of not being limited by such constraints.

It has been known that cells after receiving bearable mechanical damages can repair themselves^28^, and punctured cell membranes can recover due to their fluidity and dynamic structure^29^. In the present experiments, different oscillation frequencies were tested and an effective condition for cell transfection was identified to be 100 Hz and 10 volt amplitude for 4 mins, since under such a condition, cell death is not significantly affected and the time allowed for transfection is reasonably long. The MTT results in Fig. 2 show that the mechanical oscillations increased the membrane permeability, so that small particles such as C60 molecules and polystyrene beads penetrated into the cells much easier than larger particles, thus producing higher cytotoxicity. The results also suggest that after subjecting to mechanical oscillations, small objects like oligonucleotides may penetrate through the cell membrane easily since most of the oligonucleotides are around 22 bp, which corresponds to 7.5 nm in length and 2 nm in diameter^30^,^31^,^32^. Besides, the results in Fig. 2 also indicate that larger particles can also penetrate through the cell membrane after oscillations at 100 Hz and 10 volt amplitude for 4 mins, albeit the penetration rate is lower than the smaller particles.

SiRNAs interference has been a widely used technique in gene silencing^33^ as a therapeutic strategy to tackle cancer and other diseases^34^. In the present work, a well-designed actin-targeted siRNAs sequence was employed to demonstrate the transfection effect of mechanical oscillations. Since there are many unknown components in the Fetal Bovine Serum (FBS) and some of these may interfere with the siRNAs, and also, antibiotics and antimycotics can damage the cells after membrane opening^35^, in this work, the cell culture medium was replaced by an electroporation buffer (see materials and methods section) that can prevent siRNAs interference by the FBS and cellular damage after membrane opening^36^. The mechanical oscillations may also produce a mild heating effect, thus the cells were immediately cooled down by ice to avoid heat damage, and they were then immediately cultured in the 1640 culture medium with 10% FBS without antibiotic and antimycotic components. Figure 3 clearly shows that the siRNAs can be transfected into the K562 cells by mechanical oscillations, and Fig. 4 indicates that they were functional in interfering the translation of the actin proteins, resulting in weakening of the actin cytoskeleton. From Fig. 3 we can see that the transfection efficiency is around 30%, which is dependent on the oscillation condition in particular the duration. A high transfection efficiency up to 100% can be achieved if the intensity or duration of the vibration increases, but unavoidably this may lead to increased cell death. This is similar to electroporation, where a higher power and longer duration would result in higher transfection efficiency but also higher cell death rate^37^.

## Concluding Remarks

Mechanical oscillations at appropriate frequencies and intensities can effectively induce gene-material transfection into suspended cells, without affecting their viability. This method can be used to deliver nano-materials into targeted cells for biomedical research and for combating cancer and diseases. In addition to non-adherent cells, this method may also be applicable to adherent cells, since most of the adherent cells can be trypsinized into suspended cells. The application potential of this method for nano-material/oligonucleotide delivery is therefore high.

## Materials and Methods

### Cell culture and cell death determination

Myelogenous leukemia cell line K562 was cultured with RPMI 1640 culture medium, supplemented with 10% fetal bovine serum and 1% antibiotic and antimycotic solution (Sigma, USA), in a cell incubator (Bionex, Model-VS-9160C, Korea) filled with 5% CO_2_ at 37 °C and 95% humidity. The culture medium was changed every 48 hours. Well cultured cells were filled into 1.5 ml EP tubes which were fixed onto the motion bar of a Sine Wave Generator (PASCO, USA) (see supplementary Figure S1), which oscillated the samples at frequencies 0, 10, 100, 500 and 800 Hz, within a 10 volt amplitude input, for different durations (1, 2, 4, 8, 12 and 16 mins). The oscillated cells were stained with Trypan Blue and measured using hemocytometry. A batch of cells vibrated for 4 mins was also cultured for 1, 3 and 7 days separately and the death rates were also measured by the same method.

### Nanoparticles transfection and MTT assay measurement

In order to measure the particle delivery effect under mechanical oscillations, C60 molecules (SES Research, USA) and nano- and micro-polystyrene beads (57 nm, 120 nm, 220 nm, 390 nm and 500 nm) (Bangs Laboratories, USA) were mixed separately with K562 cells at 10^6^ cells/ml in a final concentration of 20 μg/ml and oscillated at 100 Hz and 10 V amplitude for 4 mins. The oscillated particle-mixed cells were cultured for 0 and 48 hours in the 96-well microplates and measured using the MTT (3-(4,5-dimethylthiazol-2-yl)-2,5-diphenyltetrazolium bromide) assay. The microplates were read in absorbance at 570 nm wavelength in an Asys UVM 340 microplate reader (Biochrom, UK).

### SiRNAs Transfection

Actin siRNAs (sequence: 5′-CCAGCACCAUGAAGAUCAA dTdT-3′) with FITC labelled (Thermofisher, USA) and negative control siRNAs (sequence: 5′-GGCTACGTCCAGGAGCGCA-3′) (GE Healthcare dharmacon, USA) were used to evaluate the transfection efficiency of the mechanical oscillations. The siRNAs were mixed with K562 cells in an electroporation transfection buffer (HEPES 21 mM, NaCl 137 mM, KCl 5 mM, Na_2_HPO_4_-7H_2_O 0.7 mM, Dextrose 6 mM), with the purpose to avoid interfering by the culture media, and the samples were allowed to settle for 5 mins before use. The final concentration of the actin siRNAs was 100 nM. The siRNAs mixed K562 cells were moved into EP tubes and oscillated by the PASCO Sine Wave Generator at 0 and 100 Hz and 10 V amplitude for 4 mins. After that, the EP tubes containing the siRNAs mixed cells were put on top of ice for 5 mins, and then the cells were immediately diluted 10-fold in a non-antibiotic and non-antimycotic containing culture medium, for culture in a 24-well plate for 24 hours.

### Flowcytometry

FITC labelled siRNAs transfected K562, THP-1, OCI-AML3 leukemia cells and trypsinized Hela cells were replenished with fresh, normal culture medium and stained with PI for half an hour; the stained cells were brought to the flowcytometry instrument for measurement (BD FACSAria SORP cell sorter, USA).

### Western Blot

K562 cells mixed respectively with actin siRNAs and the negative control siRNAs were exposed to mechanical oscillations at 100 Hz and 10 V amplitude for 4 mins. The cells were cultured in a 6-well plate for 24 hours, and then harvested in a SDS-protease inhibitor buffer (65 mM Tris-Cl pH 6.8, 10% glycerol, 2% SDS, 1 mM sodium orthovanadate and 1 mM sodium fluoride, 1 μg/ml aprotinin, 1 μg/ml leupeptin, 1 μg/ml pepstatin A, 1 mM phenylmethyl sulfonyl) and quantified using a DC protein assay kit (Bio-Rad, USA). Same amounts of proteins were isolated by the SDS-polyacrylamide gels and transferred to a nitrocellulose membrane. The membrane was blocked overnight at 4 °C and incubated with the primary antibodies anti-β-actin (1:2000) (Sigma, USA) and anti-GAPDH (1:500) (Pierce, USA), respectively. The primary protein labelled membrane was then probed with peroxidase-conjugated anti-rabbit and anti-mouse secondary antibodies respectively (1:3000) (Bio-Rad, USA) for 1 h, and finally detected by an ECL chemiluminescence reagent (Perkin-ELMER, USA) and exposed onto X-ray films.

### Optical Tweezers indentation

K562 cells transfected respectively with actin siRNAs and the negative control siRNAs by mechanical oscillations were cultured in fibronetin protein pre-coated confocal dishes for 24 hours. The dishes cultured with cells were brought to an Optical Tweezers system (MMI, Switzerland) for cell indentation. The cells were indented following the same protocol in our previous study^18^.

### Statistical analysis

The death proportions of K562 cells exposed to mechanical oscillations as measured by hemocytometry, MTT assay and flow cytometry were analyzed by two-tailed Chi-square tests.

### Finite-element analysis

Details are given in the Supplementary Material.

## Additional Information

**How to cite this article**: Zhou, Z. L. *et al.* Mechanical oscillations enhance gene delivery into suspended cells. *Sci. Rep.* **6**, 22824; doi: 10.1038/srep22824 (2016).

## Supplementary Material

Supplementary Information

Supplementary Movie

## References

[b1] Felgner P. L. *et al.* Lipofection: a highly efficient, lipid-mediated DNA-transfection procedure. Proc Natl Acad Sci USA 84, 7413–7417 (1987). 2823261 10.1073/pnas.84.21.7413 PMC299306

[b2] Putnam D. Polymers for gene delivery across length scales. Nat Mater 5, 439–451 (2006). 16738681 10.1038/nmat1645

[b3] Jiang M. & Chen G. High Ca2+-phosphate transfection efficiency in low-density neuronal cultures. Nat Protoc 1, 695–700 (2006). 17406298 10.1038/nprot.2006.86

[b4] Rahim A. A. *et al.* Efficient gene delivery to the adult and fetal CNS using pseudotyped non-integrating lentiviral vectors. Gene Ther 16, 509–520 (2009). 19158847 10.1038/gt.2008.186

[b5] He W. *et al.* Discovery of siRNA lipid nanoparticles to transfect suspension leukemia cells and provide *in vivo* delivery capability. Mol Ther 22, 359–370 (2014). 24002693 10.1038/mt.2013.210 PMC3916034

[b6] Karra D. & Dahm R. Transfection techniques for neuronal cells. J Neurosci 30, 6171–6177 (2010). 20445041 10.1523/JNEUROSCI.0183-10.2010 PMC6632737

[b7] Kim T. K. & Eberwine J. H. Mammalian cell transfection: the present and the future. Anal Bioanal Chem 397, 3173–3178 (2010). 20549496 10.1007/s00216-010-3821-6 PMC2911531

[b8] Capecchi M. R. High efficiency transformation by direct microinjection of DNA into cultured mammalian cells. Cell 22, 479–488 (1980). 6256082 10.1016/0092-8674(80)90358-x

[b9] Cuerrier C. M., Lebel R. & Grandbois M. Single cell transfection using plasmid decorated AFM probes. Biochem Bioph Res Co 355, 632–636 (2007). 10.1016/j.bbrc.2007.01.190 17316557

[b10] von der Leyen H. E. *et al.* A pressure-mediated nonviral method for efficient arterial gene and oligonucleotide transfer. Hum Gene Ther 10, 2355–2364 (1999). 10515455 10.1089/10430349950017004

[b11] Mann M. J. *et al.* Pressure-mediated oligonucleotide transfection of rat and human cardiovascular tissues. P Natl Acad Sci USA 96, 6411–6416 (1999). 10.1073/pnas.96.11.6411 PMC26895 10339601

[b12] Shimizu K. *et al.* *In vivo* Site-Specific Transfection of Naked Plasmid DNA and siRNAs in Mice by Using a Tissue Suction Device. Plos One 7 (2012). 10.1371/journal.pone.0041319 PMC3402481 22844458

[b13] Neumann E., Schaeferridder M., Wang Y. & Hofschneider P. H. Gene-Transfer into Mouse Lyoma Cells by Electroporation in High Electric-Fields. Embo J 1, 841–845 (1982). 6329708 10.1002/j.1460-2075.1982.tb01257.x PMC553119

[b14] Zhao Y. *et al.* High-efficiency transfection of primary human and mouse T lymphocytes using RNA electroporation. Mol Ther 13, 151–159 (2006). 16140584 10.1016/j.ymthe.2005.07.688 PMC1473967

[b15] Husaini A. M. *et al.* Vehicles and ways for efficient nuclear transformation in plants. GM Crops 1, 276–287 (2010). 21844685 10.4161/gmcr.1.5.14660

[b16] Ng K. S., Zhou Z. L. & Ngan A. H. W. Frequency-dependent cell death by optical tweezers manipulation. J Cell Physiol 228, 2037–2041 (2013). 23553530 10.1002/jcp.24369

[b17] Fu P. P., Xia Q., Hwang H. M., Ray P. C. & Yu H. Mechanisms of nanotoxicity: generation of reactive oxygen species. J Food Drug Anal 22, 64–75 (2014). 24673904 10.1016/j.jfda.2014.01.005 PMC9359151

[b18] Zhou Z. L., Hui T. H., Tang B. & Ngan A. H. W. Accurate measurement of stiffness of leukemia cells and leukocytes using an optical trap by a rate-jump method. Rsc Adv 4, 8453–8460 (2014).

[b19] Rawicz W., Olbrich K., McIntosh T., Needham D. & Evans E. Effect of chain length and unsaturation on elasticity of lipid bilayers. Biophys J 79, 328–339 (2000). 10866959 10.1016/S0006-3495(00)76295-3 PMC1300937

[b20] Clark A. G., Dierkes K. & Paluch E. K. Monitoring actin cortex thickness in live cells. Biophys J 105, 570–580 (2013). 23931305 10.1016/j.bpj.2013.05.057 PMC3736691

[b21] Dumont F., Marechal P.-A. & Gervais P. Cell size and water permeability as determining factors for cell viability after freezing at different cooling rates. Appl Environ Microbiol 70, 268–272 (2004). 14711651 10.1128/AEM.70.1.268-272.2004 PMC321282

[b22] Fischer T. M. Bending stiffness of lipid bilayers. I. Bilayer couple or single-layer bending? Biophys J 63, 1328 (1992). 1477282 10.1016/S0006-3495(92)81710-1 PMC1261437

[b23] Campillo C. *et al.* Mechanics of membrane–cytoskeleton attachment in Paramecium. New J Phys 14, 125016 (2012).

[b24] Mehier-Humbert S. & Guy R. H. Physical methods for gene transfer: Improving the kinetics of gene delivery into cells. Adv Drug Deliver Rev 57, 733–753 (2005). 10.1016/j.addr.2004.12.007 15757758

[b25] Marsh M. & McMahon H. T. The structural era of endocytosis. Science 285, 215–220 (1999). 10398591 10.1126/science.285.5425.215

[b26] Gao X. & Huang L. Cationic liposome-mediated gene transfer. Gene Ther 2, 710–722 (1995). 8750010

[b27] Burns J. C., Friedmann T., Driever W., Burrascano M. & Yee J. K. Vesicular Stomatitis-Virus G Glycoprotein Pseudotyped Retroviral Vectors - Concentration to Very High-Titer and Efficient Gene-Transfer into Mammalian and Nonmammalian Cells. P Natl Acad Sci USA 90, 8033–8037 (1993). 10.1073/pnas.90.17.8033 PMC47282 8396259

[b28] Miyake K. & McNeil P. L. Mechanical injury and repair of cells. Crit Care Med 31, S496–S501 (2003). 12907878 10.1097/01.CCM.0000081432.72812.16

[b29] Tovar O. & Tung L. Electroporation and Recovery of Cardiac Cell-Membrane with Rectangular Voltage Pulses. Am J Physiol 263, H1128–H1135 (1992). 1415761 10.1152/ajpheart.1992.263.4.H1128

[b30] Smith S. B., Cui Y. & Bustamante C. Overstretching B-DNA: the elastic response of individual double-stranded and single-stranded DNA molecules. Science 271, 795–799 (1996). 8628994 10.1126/science.271.5250.795

[b31] Hansen K. M. *et al.* Cantilever-based optical deflection assay for discrimination of DNA single-nucleotide mismatches. Anal Chem 73, 1567–1571 (2001). 11321310 10.1021/ac0012748

[b32] Schroeder A., Levins C. G., Cortez C., Langer R. & Anderson D. G. Lipid-based nanotherapeutics for siRNA delivery. J Intern Med 267, 9–21 (2010). 20059641 10.1111/j.1365-2796.2009.02189.x PMC5308083

[b33] Meister G. & Tuschl T. Mechanisms of gene silencing by double-stranded RNA. Nature 431, 343–349 (2004). 15372041 10.1038/nature02873

[b34] Devi G. R. siRNA-based approaches in cancer therapy. Cancer Gene Ther 13, 819–829 (2006). 16424918 10.1038/sj.cgt.7700931

[b35] Lewis K. Platforms for antibiotic discovery. Nat Rev Drug Discov 12, 371–387 (2013). 23629505 10.1038/nrd3975

[b36] Huang Y. Y. *et al.* Elimination Pathways of Systemically Delivered siRNA. Mol Ther 19, 381–385 (2011). 21119623 10.1038/mt.2010.266 PMC3034859

[b37] Geng T., Zhan Y. H., Wang J. & Lu C. Transfection of cells using flow-through electroporation based on constant voltage. Nat Protoc 6, 1192–1208 (2011). 21799488 10.1038/nprot.2011.360

