# Supplementary material for: Mechanical oscillations enhance gene delivery into suspended cells
Source: Sci Rep. 2016 Mar 9;6:22824. doi: 10.1038/srep22824 (PMC4783776; doi:10.1038/srep22824)
Supplement: Supplementary Information [file srep22824-s1.pdf]

Mechanical oscillations enhance gene delivery into suspended cells

Z.L. Zhou, X.X. Sun, J. Ma, C.H. Man, A.S.T Wong,

A.Y. Leung and A.H.W. Ngan

Supplementary Material

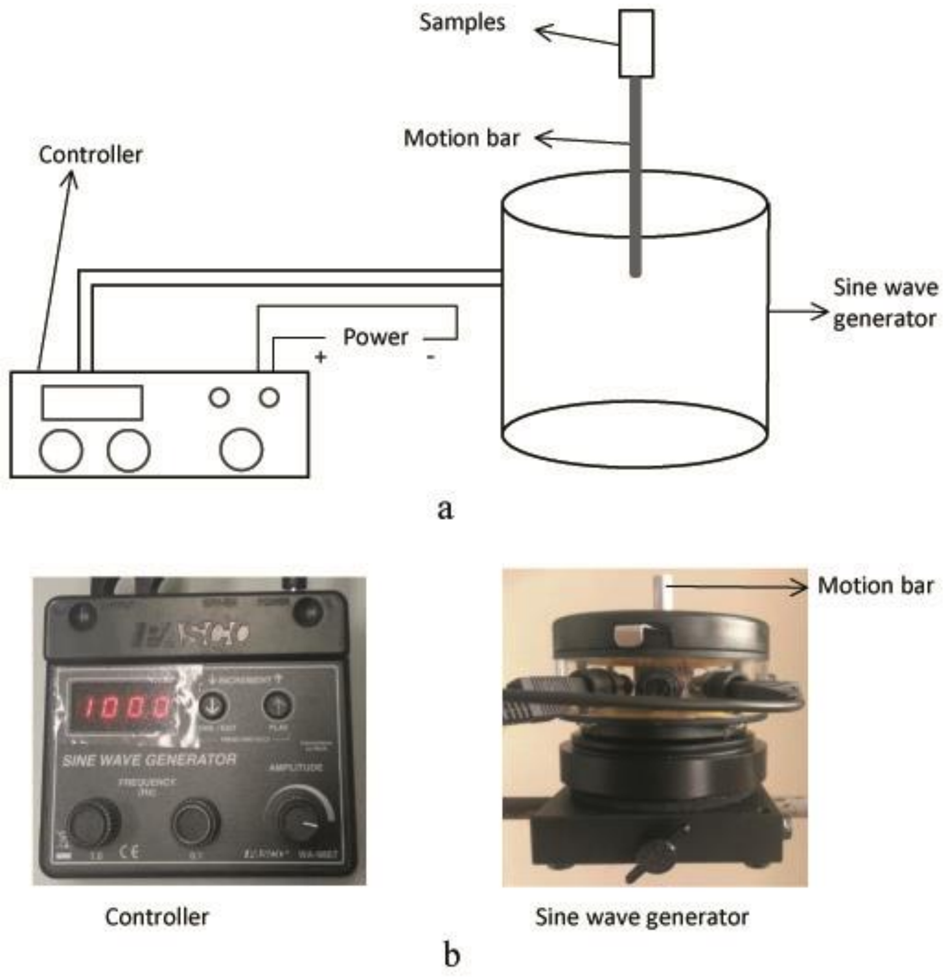

Figure S1. PASCO Sine Wave Generator

Figure S1. K562 cells were filled into 1.5 ml EP tubes which were fixed onto the motion bar of a Sine Wave Generator (PASCO, USA), which oscillated the samples at frequencies 0, 10, 100,500 and 800 Hz, within a 10 volt amplitude input, for different durations (1, 2, 4, 8, 12 and 16 mins).

Scanning Electronic Microscope observation

K562 cells were mixed with C60 molecules and nano- and micro- polystyrene beads separately and oscillated at 100 Hz and 10 V amplitude for 4 mins. The oscillated particle-mixed cells were then cultured in a 24-well culture plates which was pre-inserted by sterile coverslips (14mm in diameter) for 24 hours. The remaining procedure followed the protocol in our previous study [1]. The particle mixed cells were fixed with 2.5% glutaraldehyde in 0.1 M cacodylate buffer for 2 h at 4 °C. The cells were dehydrated for 5 min in a series of ethanol solutions of increasing concentration (30%, 50%, 75%, 90% and 100%). The samples were then dried in a critical point dryer before examination in a scanning electron microscope (Hitachi S4800 FEG SEM, Japan) without coating with gold nanoparticles.

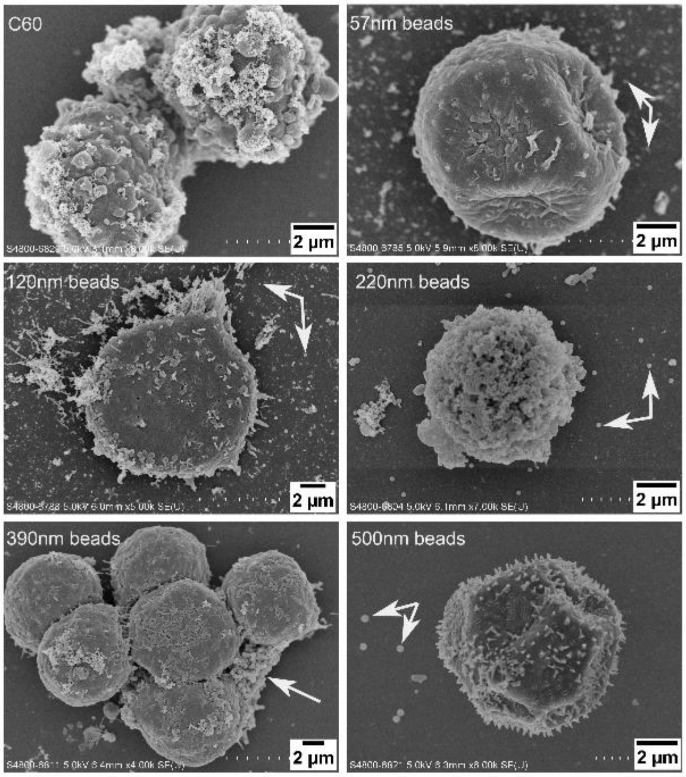

Figure S2. SEM images of K562 cells mixed with particles of different sizes after subjecting to mechanical oscillations at 100 Hz and 10 V amplitude for 4 mins. White arrows indicate the particles. The nano- and micro- particles can be observed under SEM, as indicated by the white arrows in Figure 2b, although a single C60 molecule is around 1 nm [2] and is therefore not observable under the SEM.

Finite-element model for the cytoskeleton cortex

To theoretically investigate how the cortex structure can be disrupted by vibrations, finite-element simulation was performed. The cortex layer of the cytoskeleton is modeled as a layer of strut-element network of thickness $0.2\mu m$ [3] on the surface of a sphere of radius $8\mu m$ [4], according to what is known about typical leukemia cells. To construct the filament network of the cortex, 28000 points were generated randomly within the shell-volume of the cortex layer from a uniform distribution, and then any two points separated by a distance between 0.25 μm and 0.35 μm [5,6] were connected up to form a strut element that represents an actin filament. In this way, the average length of the actin filaments in the cortex structure is 0.3 μm. As shown in Figure 6(a) in the main text, pores of various sizes appear randomly on the cortex layer, due to the finite thickness of the cortex layer and the random alignments of the actin filaments inside. Although actin filaments are usually modelled as a semi-polymer [7], they are considered to be straight within the cortex layer as their length of ~0.3 μm is much smaller than the persistence length of ~15 μm [8]. Therefore, in the present model, actin filaments are described as Euler beam elements with their bending modeled. In addition, the force (f)-extension (δ) relation of an actin filament is also influenced by thermal fluctuations as described by MacKintosh [9] as

$$\delta = \frac{l^2}{l_p}[\frac{1}{6} - \frac{l^2}{90 l_p k_B T} f + O(f^2)] \tag{1}$$

where l_p is the persistence length of 15 μm, $\kappa = l_p k_B T$ is the bending stiffness of 2×10^{-26} Nm^2 [10], and l is the length of an actin filament. As the length of the actin filament in this model is very short compared with its persistence length, for small axial deformations, the higher terms $O(f^2)$ in eqn. (1) is $\sim 2.6 \times 10^{-4}$ which is orders of magnitude smaller than the first two terms. Therefore, the force-extension relation for the actin filament is effectively linear. Based on eqn. (1), an effective Young's modulus can be derived and used for the beam element in our simulation as

$$E_{eff} = \frac{90 l_p \kappa}{A\, l^3} \tag{2}$$

where A is the cross-sectional area of an actin filament, the diameter of which is $7\ nm$ [7]. Then the cortex structure was set into motion by oscillating two nodal points one on the top and one on the bottom of the structure in a horizontal (tangential) direction according to the following displacement function

$$X(t) = 0.2\sin(200\pi t) \quad [\mu m] \tag{3}$$

where t is time in seconds, i.e. the oscillation frequency is 100 Hz, and amplitude 0.2 µm, which replicate well the experimental conditions. The mass density of actin filaments is on the order of 810 Daltons/μm^3, where 1 Dalton = 1.66x10^{-24}g, so this is assigned be ~ $10^{-12} g/\mu m^3$ [11]. The cortex is connected to other intracellular components through the cytoskeleton, essentially microtubules. To represent the effect of the inertia of the intracellular components on the oscillation, the mass of the intracellular components is equivalently distributed onto the cortex though these microtubules connections. It has been reported that in nerve axons, the density of microtubules on the cortex is about $100/\mu m^2$ [12]. However, in a leukemia cell, which is of the suspension type, the density of microtubules should be lower than this value for nerve axons. In our simulation, therefore, ~800 mass points representing the microtubule connections are distributed on the cortex structure, the summation of which is equal to $4 \times 10^{-9} g$ which is the approximate mass of a cell [13].

To quantify the disruptions of the cortex structure due to the vibrations, we analyzed the statistical distribution of the areas of the pores existing on the cortex layer. The software CloudCompare for 3D point cloud and mesh processing [14] was used. The nodal coordinates of the cortex structure before and after vibration were imported into the software, which output information about the cortex structure based on node density. When the node density is lower than a certain threshold value, a pore is assumed to exist, and by scanning the cortex structure against this threshold density, we can figure out the position and shape of the pores. Then the areas of the pores were calculated by counting the number of a set of points deposited onto them from a uniform density. The simulated oscillations can be seen from the movie attached.

References

1 Zhou, Z. L. *et al.* AFM nanoindentation detection of the elastic modulus of tongue squamous carcinoma cells with different metastatic potentials. *Nanomed-Nanotechnol* **9**, 864-874 (2013).

2 Goel, A., Howard, J. B. & Vander Sande, J. B. Size analysis of single fullerene molecules by electron microscopy. *Carbon* **42**, 1907-1915 (2004).

3 Clark, A. G., Dierkes, K. & Paluch, E. K. Monitoring actin cortex thickness in live cells. *Biophys J* **105**, 570-580 (2013).

4 Dumont, F., Marechal, P.-A. & Gervais, P. Cell size and water permeability as determining factors for cell viability after freezing at different cooling rates. *Appl Environ Microbiol* **70**, 268-272 (2004).

5 Chaudhuri, O., Parekh, S. H. & Fletcher, D. A. Reversible stress softening of actin networks. *Nature* **445**, 295-298 (2007).

6 Gowrishankar, K. *et al.* Active remodeling of cortical actin regulates spatiotemporal organization of cell surface molecules. *Cell* **149**, 1353-1367 (2012).
7 Mofrad, M. R. & Kamm, R. D. *Cytoskeletal mechanics: models and measurements in cell mechanics*. (Cambridge University Press, 2006).
8 Salbreux, G., Charras, G. & Paluch, E. Actin cortex mechanics and cellular morphogenesis. *Trends Cell biol* **22**, 536-545 (2012).
9 MacKintosh, F., Käs, J. & Janmey, P. Elasticity of semiflexible biopolymer networks. *Phys Rev Lett* **75**, 4425 (1995).
10 Oosawa, F. The flexibility of F-actin. *Biophys Chem* **11**, 443-446 (1980).
11 Bremer, A. *et al.* The structural basis for the intrinsic disorder of the actin filament: the" lateral slipping" model. *J Cell Biol* **115**, 689-703 (1991).
12 Thies, E. & Mandelkow, E.-M. Missorting of tau in neurons causes degeneration of synapses that can be rescued by the kinase MARK2/Par-1. *J Neurosci* **27**, 2896-2907 (2007).
13 Park, K. *et al.* 'Living cantilever arrays' for characterization of mass of single live cells in fluids. *Lab Chip* **8**, 1034-1041 (2008).
14 *CloudCompare (version 2.6)*, <http://www.cloudcompare.org/> (2015).
